# Increased contact activated endogenous thrombin potential in pregnant women with preeclampsia

**DOI:** 10.1097/MBC.0000000000001269

**Published:** 2023-11-30

**Authors:** Anne Cathrine Godtfredsen, Yaseelan Palarasah, Britta Blume Dolleris, Jan Stener Jørgensen, Johannes Jakobsen Sidelmann, Jørgen Brodersen Gram

**Affiliations:** a Department of Gynecology and Obstetrics, University Hospital of Southern Denmark; b Unit for Thrombosis Research, Department of Regional Health Research, University of Southern Denmark, Esbjerg; c Department of Cancer and Inflammation Research, University of Southern Denmark; d Department of Gynecology and Obstetrics, University Hospital of Southern Denmark, Odense; e Department of Clinical Biochemistry, University Hospital of Southern Denmark, Esbjerg, Denmark

**Keywords:** coagulation, contact system, preeclampsia, pregnancy, thrombin generation, tissue factor

## Abstract

Preeclampsia is a worldwide contributor to maternal and fetal morbidity and mortality. Women with preeclampsia are in a hyper-coagulable state with increased risk of thromboembolic disease later in life compared with normal pregnant women. The contact system (CAS) in plasma can mediate thrombin generation and is an important contributor to thrombus growth, but the activation of CAS during pregnancy complicated by preeclampsia is not yet elucidated, and CAS may play a role in the pathophysiology of preeclampsia. Therefore, the aim of the study is to address thrombin generation, and in particular, the capacity of the CAS-mediated pathway in patients with preeclampsia compared with pregnant controls. One hundred and seventeen women with preeclampsia and matched controls were included. The project was registered at www.clinicaltrials.gov as NCT04825145. CAS and tissue factor induced thrombin generation, proteins C and S, antithrombin, and histidine-rich glycoprotein (HRG) were assessed. Women with preeclampsia had significantly increased CAS and tissue factor-induced endogenous thrombin potential (ETP), and HRG compared with controls, *P* = 0.022, *P* = 0.024, and *P* = 0.02, respectively. The concentrations of protein C and antithrombin were significantly reduced in the preeclampsia group, *P* = 0.024 and *P* < 0.0001, respectively. No significant difference in the concentration of protein S was detected, *P* = 0.06. This study demonstrates a significant increased CAS-induced ETP and an overall decrease of important regulators of coagulation in women with preeclampsia compared with controls. These aspects can contribute to the hyper-coagulable state characterizing preeclampsia.

**KEY MESSAGE**

- This study demonstrates a significant increased contact system and tissue factor-induced ETP, increased HRG, and decrease of important regulators of coagulation in pregnant women with preeclampsia compared with controls.

## Introduction

Preeclampsia is a pregnancy-specific systemic disorder and can cause both maternal and fetal morbidity and mortality. Approximately 5% of pregnancies are complicated by preeclampsia [1]. Most clinical guidelines define preeclampsia as new onset of hypertension after gestational week 20 accompanied by proteinuria or other organ dysfunctions [2,3]. The disease has been known for centuries, but the pathogenesis is still not fully understood. Both placental and maternal conditions probably contribute to the development of the disease. Predisposition to endothelial dysfunction is thought to play a central role [4] and can lead to inflammation and hemostatic abnormalities. Women with preeclampsia are in a hyper-coagulable state compared with normal pregnant women [5], and clinical features of the disease are associated with intravascular coagulation [6], which can also complicate preeclampsia. Furthermore, women with a history of preeclampsia have an increased risk of future cardiovascular disease such as ischemic heart disease, stroke, and venous thromboembolism [1,4]. In addition, overweight, renal disease, chronic hypertension, and diabetes are all risk factors for preeclampsia and for the future risks mentioned above.

Hemostasis can be influenced by many factors [7]. Simplified, the two coagulation pathways leading to thrombin generation are either tissue factor-dependent or surface-induced [8]. Thrombin is one of the most important proteases of the coagulation system, cleaving fibrinogen to fibrin, the endpoint of coagulation. The tissue factor-dependent pathway is activated by trauma leading to tissue factor release. Tissue factor activates coagulation factor VII (FVII) to activated FVII that triggers the common pathway of coagulation leading to the formation of thrombin. The surface-induced pathway is initiated by the contact system (CAS), by activating coagulation factor XII (FXII) to activated FXII (FXIIa). The interaction of CAS and an activating surface determines the propagation of CAS [9], and may lead to initiation of coagulation, fibrinolysis or inflammation [10]. FXIIa can activate coagulation factor XI (FXI) to an activated form (FXIa) and proceed to the common pathway of coagulation resulting in thrombin formation. Changes in the concentration or activity of FXII are not related to hemostasis, but support the fibrin-forming mechanism on the platelet-rich thrombus during thrombosis [11]. CAS and FXII can hereby contribute to thrombus growth and by that to the pro-thrombotic state characterizing women with preeclampsia.

Although the tissue factor pathway of thrombin generation has been studied previously [12–16] the knowledge on contact-induced thrombin generation is sparse in preeclampsia. The aim of the present cross-sectional study is to address thrombin generation, and in particular the capacity of the CAS-mediated pathway in patients with preeclampsia compared with pregnant controls.

## Methods

### Cohort

The patient cohort is described previously [10].

The study is a matched cross-sectional trial conducted from January 2020 to October 2021. Patients were included at Departments of Obstetrics and Gynecology at University Hospital of Southern Denmark, Esbjerg, and Odense.

Inclusion of patients with preeclampsia were based on the definition in the National Guideline from 2018 [2] as a combination of hypertension and proteinuria after 20 weeks of gestation or hypertension accompanied by one of the following: hematological or neurological complications, liver dysfunction, renal failure, pulmonary edema or utero placental insufficiency. The National Guideline is in overall accordance with the ‘International Society for the Study of Hypertension in Pregnancy’ (ISSHP) guideline from 2018 [3]. Pregnant women above the age of 18 fulfilling the diagnostic criteria of PE were enrolled in the study. One-hundred and seventeen women with preeclampsia accepted to participate.

For each woman with preeclampsia, a healthy pregnant woman was included. The control subjects were matched with cases with respect to gestational age (±1 week), age (±1 year) and pregestational BMI (±1 kg/m^2^). One thousand and forty-four pregnant women consented to be possible controls. Pregnant women with the best match on all three criteria were telephoned and invited to participate, having their blood sample taken at the matched gestational age. Overall, 234 pregnant women were enrolled in the study. Data concerning age, parity, smoking status, medication during pregnancy, medical and obstetrical history, blood pressure, laboratory results, gestational age at delivery, delivery method, survival, birth weight, sex and maternal together with fetal complications were collected from their medical records.

### Blood sampling

We collected blood samples from the patients at inclusion upon establishment of the preeclampsia diagnosis and from the controls in a comparable gestational week. The collection and handling of blood specimens followed the G41 guideline from Clinical and Laboratory Standards Institute (CLSI) [17] and the H21-A5 guideline from CLSI [18].

We collected 16.8 ml blood from an antecubital vein in four evacuated 2.7 ml tubes containing 0.105 mol/l sodium citrate (Vacutainer 9NC, Becton Dickinson, Plymouth, UK) and two evacuated 3 ml tubes containing 5.4 mg dipotassium-ethylene-diamine-tetraacetate (EDTA) (Vacutainer K2E, Becton Dickinson). Platelet poor plasma was collected after centrifugation for 20 min at 2000*g*. The citrate and EDTA-stabilized plasma samples were subsequently stored at −80 °C in tightly capped cryotubes (Sarstedt, VWR-Bie & Berntsen, Søborg, Denmark). All samples were stored in a biobank. Before analysis, the samples were thawed for 5 min at 37°C, kept at room temperature, and analyzed within one hour.

### Biochemical methods

Tissue factor-induced thrombin generation was analyzed by the calibrated automated thrombin generation assay (Thrombinoscope BV, Maastricht, The Netherlands) [19]. In brief, citrate-stabilized plasma was activated by tissue factor in the presence of phospholipids and the concentration of thrombin generated as a function of time, was recorded using a fluorogenic substrate employing the Fluoroskan Ascent microplate fluorometer from Thermo Fisher Scientific (Hvidovre, Denmark). The Thrombinoscope software was used for calculation of the various measures of thrombin generation: the lag time of the thrombin formation process, the time the peak thrombin concentration was reached (time to peak), the peak thrombin concentration and the endogenous thrombin potential (ETP) recording the total amount of thrombin formed. The inter-serial coefficient of variation ranged from 3.6 to 6.7% on the various measures of thrombin generation.

The contact activated thrombin generation was analyzed by a combination of the kallikrein generation assay described elsewhere [20] and the tissue factor-induced thrombin generation assay. In brief, 100 μl STA-PTT from Diagnostica Stago (Asnieres-sur-Seine, France) was mixed with 100 μl of Silica Ludox AS-40 from Sigma-Aldrich (Darmstadt, Germany) (375 μl Silica AS-40 + 4625 μl H_2_O). Subsequently, 60 μl of STA-PTT/Silica was mixed with 525 μl of BSA60 buffer [20]. The measures of thrombin generation were determined as described for tissue factor-induced thrombin generation. The inter-serial CV ranged from 4 to 8.4% on the various measures of thrombin generation.

Plasma-free protein S antigen was determined using an immuno-turbidimetric assay kit (HemosIL, Instrumentation Laboratory, Bedford, USA) employing the ACLTOP350 Coagulation Analyzer. Inter-serial CV was 4.2%.

Plasma protein C antigen was determined by an in-house enzyme immunoassay (ELISA) with antibodies from Cedarlane/Affinity Biologicals (Burlington, Canada/Ancaster, Ontario, Canada). Inter-serial CV was 6.1%.

Plasma antithrombin was determined using an immunochemical assay kit and nephelometer (Behring Nephelometer Analyzer II, Siemens Healthcare Dignostics Products GmbH, Marburg, Germany). Inter-serial CV was 3.7%.

The protein concentration of C1 esterase inhibitor (C1-inh) was determined using N antiserum against human C1inh, buffers, and reagents, using the BN II analyser (all from Siemens Healthcare Diagnostics, Marburg, Germany). Inter-serial CV was 1.6%.

HRG was determined with the Human HRG ELISA Kit from Abcam (Cambridge, UK). Inter-serial CV was less than 12%.

### Statistical analyses

We used GraphPad Prism version 9.1.2 (GraphPad Software, San Diego, California, USA) for statistical calculations. A QQ plot and the Kolmogorov–Smirnov test verified the distribution of the results. Paired *t* test, Wilcoxon matched-pairs signed rank test, unpaired *t* test, Mann–Whitney, or Chi^2^ test were applied as appropriate. Associations between variables were assessed by the Pearson product moment correlation analyzes. Possible confounders were adjusted for by using multiple linear regression.

Results are presented as mean ± standard deviation or median and 25–75 percentile range, as appropriate. A *P* value less than 0.05 was considered statistically significant.

### Ethics statement

Informed written consent was obtained from the participants before inclusion. The study was conducted according to the Helsinki declaration including study approval by the Danish Data Protection Agency. Data from study participants are protected according to the Act on Processing of Personal Data. The Regional Ethics Committee in the Region of Southern Denmark approved the project (project ID S-20190142, 10 December 2019). The project was registered at www.clinicaltrials.gov as NCT04825145.

## Results

Women with preeclampsia were comparable with controls with respect to the matching criteria, maternal age, pregestational BMI, and gestational age.

The characteristics of the study participants are shown in Table 1.

**Table 1:** Characteristics of the study participants

	Women with preeclampsia (*n* = 117)	Controls (*n* = 117)	*P* value
Pregnancy (number)	2 (1–9)	2 (1–11)	0.15
Nullipara	56.4%	44.4%	0.07
Current pregnancy Singleton/multifetal	93.2% singleton 6.8% gemelli	96.6% singleton 3.4% gemelli	0.24
Blood pressure in early pregnancy (mmHg)	Systolic: 124 ± 12 Diastolic: 81 ± 10	Systolic: 117 ± 12 Diastolic: 75 (48–96)	<0.0001
Birthweight (g)	2992 ± 688	3612 ± 549	<0.0001
Gestational age at delivery	38 + 2(30 + 1 − 42 + 0)	40 + 3(35 + 5 – 42 + 3)	<0.0001
Assisted reproductive treatment	12.0%	9.5%	0.54
Smoking status	81.2% nonsmokers 12.8% former smokers 6.0% current smokers	91.5% nonsmokers 3.4% former smokers 5.1% current smokers	0.03
Mode of delivery (%)			<0.0001
Spontaneous vaginal delivery	6	66	
Vaginal delivery after induction	61	35	
Operative vaginal delivery (ventouse)	4	3	
Emergency cesarean section	36	7	
Planned cesarean section	10	6	

The results are presented as mean ± SD or median (min-max), as appropriate.

As demonstrated in Table 1, in early pregnancy, the blood pressure was higher in the women developing preeclampsia, than in the control women, *P* less than 0.0001. The weight of the newborn was lower, and the duration of pregnancy was shorter in women with preeclampsia compared with their controls, *P* less than 0.0001 and *P* less than 0.0001, respectively. No significant difference was seen in in the number of women who had received assisted reproductive treatment, *P* = 0.54. A significant difference was noticed in the mode of delivery, where more women in the control group had spontaneous vaginal birth compared with the women with preeclampsia, *P* less than 0.0001.

**Fig. 1:**
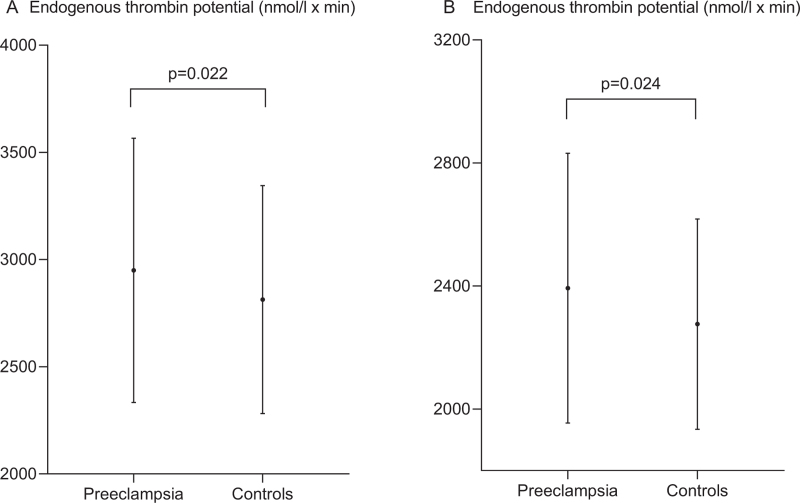
Contact system (panel A) and tissue factor induced endogenous thrombin potential (panel B).

Figure 1 demonstrates that the ETP after contact activation and tissue factor activation, were significantly higher in women with preeclampsia, compared with controls, *P* = 0.022 and *P* = 0.0024, respectively.

As shown in Table 2, the CAS induced lag time and time to peak were significantly longer in women with preeclampsia compared with controls, *P* less than 0.0001 and *P* = 0.008, respectively. The peak plasma concentration of thrombin after CAS activation was significantly higher, whereas the peak concentration after tissue factor activation was significantly lower, in the preeclampsia group compared with the control group, *P* = 0.0026 and *P* = 0.024, respectively.

**Table 2:** Contact system and tissue factor-induced thrombin generation

	Preeclampsia n = 117	Controls n = 117	p-value
CAS-induced thrombin generation			
Lagtime (min)	1.67 (0.92–2.33)	0.67 (0.50–1.67)	<0.0001
ttPeak (min)	4.8 (4.3–5.3)	4.7 (4.3–5.0)	0.008
Peak (nmol/l)	391 ± 81	375 ± 73	0.026
ETP (nmol/l × min)	2950 ± 616	2813 ± 532	0.022
TF-induced thrombin generation			
Lagtime (min)	3.0 (2.7–3.3)	3.0 (2.7–3.2)	0.54
ttPeak (min)	6.0 (5.3–7.0)	5.5 (5.0–5.8)	<0.0001
Peak (nmol/l)	366 ± 92	417 ± 58	<0.0001
ETP (nmol/l × min)	2393 ± 439	2276 ± 342	0.024

CAS, contact system; ETP, endogenous thrombin potential; TF, tissue factor; ttPeak, time to peak. The results are presented as mean ± SD or median (25–75 percentiles), as appropriate.

In the tissue factor-activated thrombin generation, no significant difference was observed in the lag time between the two groups, *P* = 0.54, whereas the time to peak, was significantly higher in women with preeclampsia compared with controls, *P* less than 0.0001.

As presented in Table 3, a significantly lower plasma concentration of protein C and antithrombin was recorded in women with preeclampsia compared with controls, *P* = 0.024 and *P* < 0.0001, respectively. No significant difference was observed in the plasma concentration of C1-inh or free protein S between the groups, *P* = 0.14 and *P* = 0.06, respectively. The concentration of HRG was significantly higher in women with preeclampsia compared with controls, *P* = 0.02.

**Table 3:** Regulators of thrombin generation

	Preeclampsia (*n* = 117)	Controls (*n* = 117)	*P* value
Protein C (IU/ml)	0.99 ± 0.24	1.06 ± 0.25	0.024
Free-protein S (%)	55 (45–64)	52 (47 – 57)	0.06
Antithrombin (g/l)	0.26 (0.23 - 0.28)	0.28 (0.26–0.30)	<0.0001
C1-esterase inhibitor (g/l)	0.17 (0.11–0.35)	0.17 (0.11–0.38)	0.14
HRG (μg/ml)	30.1 ± 16.7	26.1 ± 14.8	0.02

HRG, histidine-rich glycoprotein. The results are presented as mean ± SD or median (25–75 percentiles), as appropriate.

Correlation analyzes demonstrated significant associations between the plasma concentration of HRG and CAS-induced lag time and time to peak, *P* = 0.04 and *P* = 0.004, respectively.

## Discussion

### Main findings

The present trial is the first matched cross-sectional study addressing CAS-induced thrombin generation in women with preeclampsia. Several studies have investigated the tissue factor-mediated pathway of thrombin generation in women with preeclampsia and discovered an increase in ETP [12–16]. Here, we demonstrate a significantly increased CAS-induced ETP in women with preeclampsia compared with controls, supporting the hyper-coagulability characterizing this disease.

Pregnancy itself may cause a procoagulant state with the advantage of reducing hemorrhage during and after delivery. In patients with preeclampsia, this procoagulant state is exaggerated, and the patients become hyper-coagulable [5] with increased risk for thromboembolic complications [21]. Clinical studies have demonstrated significant associations between increased ETP and risk of first and recurrent venous thromboembolism. Moreover, patients with inherited thrombophilia are characterized by increased ETP [22]. Thus, the increased thrombin generation demonstrated in our preeclampsia patients may contribute to the increased risk of thrombosis observed in patients with preeclampsia.

Additionally, we demonstrate that the CAS-induced lag time of thrombin generation is significantly longer in women with preeclampsia compared with controls. This observation is in line with previous findings demonstrating that prolongation of the lag time is associated with the severity of preeclampsia [23] using a thrombin generation assay sensitive to activation induced by CAS [24].

The increased CAS-induced lag time in patients with preeclampsia is, however, puzzling. A prolonged lag time may in general be related to increased C1inh, the major inhibitor of CAS. But C1inh is reduced in both controls and women with preeclampsia, and the concentration of C1inh is not significantly different between the two groups [10]. These results suggest that C1inh is not responsible for the prolonged lag time observed in women with preeclampsia. HRG, however, may also regulate the activity of CAS. HRG binds to FXIIa with high affinity and inhibits CAS by this action [25]. We demonstrate that the concentration of HRG is significantly higher in women with preeclampsia than controls and we observe a significant correlation between HRG and CAS-induced lag time, suggesting that the increased HRG in women with preeclampsia contributes to the prolonged lag time observed in these women. In parallel, we observe a positive and significant correlation between the CAS-induced time to peak and HRG. These results suggest that the velocity of the CAS-induced thrombin formation is related to the increased HRG characterizing the women with preeclampsia.

Despite the delay in thrombin formation, the thrombin-generating capacity of CAS, in terms of ETP and peak thrombin concentration, is significantly higher in women with preeclampsia than controls. We have previously demonstrated that the plasma concentration of FXII and HK are comparable in our patients with preeclampsia and controls, whereas prekallikrein is lower in the patients with preeclampsia [10]. Hence, differences in the concentration of the CAS proteins are presumably not responsible for the observed differences in thrombin generation.

Notably, in line with previous studies [12–16], we demonstrate increased tissue factor-induced ETP in the patients with preeclampsia compared with controls. The tissue factor-induced lag time is not different, whereas the peak thrombin concentration is lower and the time to peak is longer in preeclampsia patients than controls. Thus, the interpretation of the measures of tissue factor-induced thrombin generation is not straightforward.

We demonstrate, however, a significant decrease in the plasma concentration of protein C in women with preeclampsia compared with controls. These results deviate from former studies, including one of the most comprehensive trials including 142 patients with preeclampsia [26] where no difference in plasma concentration of protein C was reported. Most studies performed after 2010 show, however, a decrease in plasma concentration of protein C in women with preeclampsia [27–29]. Additionally, a review from von Dadelszen *et al.*[30] in 2002 reports a gestational age-dependent decrease of protein C during pregnancy, and it can be speculated that the women have lower baseline concentrations of protein C before developing preeclampsia than women who are not affected by preeclampsia. Many studies on protein C, are evaluating the activity of the protein [28,31–33], making the comparison of results difficult with the present trial. The plasma concentration of protein S showed no difference in preeclampsia women compared with controls, in agreement with most former studies [26–28].

Moreover, in agreement with other studies, we observed decreased antithrombin in women with preeclampsia compared with controls [34,35]. It has been suggested, that decreasing levels of antithrombin is associated with clinical worsening of preeclampsia [35].

### Strengths and limitations

The strength of this study is the thoroughly matched design. In addition, the number of participants in our study is high compared with many former studies.

The cross-sectional design is a limitation, because a potential causality between, for example, the plasma concentration of HRG, protein C or antithrombin and time to peak cannot be established by this study design. A prospective study addressing the propagation and activation of CAS in patients with preeclampsia should be performed to overcome this limitation. Another limitation of the study is, the subgroups of patients with severe preeclampsia, preterm/term preeclampsia and preeclampsia complicated by fetal growth restriction are small, increasing the risk of type 2 errors.

It could be speculated that parity serves as a possible confounder, as an increase in maternal age is inevitable. The parity was not different between the two groups. We discovered that the significant results of the study remain significant after adjustment for parity. Similarly, it was noticed that smoking status differs between the women with preeclampsia and their controls. Adjustment for this potential confounder was without significant effect on the results. Furthermore, women with multiple pregnancies were included in the study. When excluding these women and their paired controls in the analysis, no changes in the results were noticed, except for protein C where statistical difference was no longer seen with a *P* value of 0.06, instead of 0.02.

Taken together, the present study reveals, that ETP induced by both the contact system and tissue factor is significantly elevated in women with preeclampsia than controls. In addition, both AT and protein C are reduced in women with preeclampsia, hereby supporting the hyper-coagulability characterizing preeclampsia. In a former study [10], we reported a decreased kallikrein generation as an expression for the depressed inflammatory pathway of CAS in women with preeclampsia compared with controls. Combined, the studies suggest a depressed inflammatory pathway but an increased CAS-mediated coagulation in women with preeclampsia compared with controls.

## Conclusion

The present study demonstrates a significant increase in CAS-induced and tissue factor-induced ETP in women with preeclampsia compared with controls. These findings are supported by significantly decreased levels of the regulators of the common pathway of coagulation, reflecting the hyper-coagulable state characterizing women with preeclampsia. Taken together, the results provide a novel assessment on how CAS propagates during pregnancy and may contribute to the pathophysiology of preeclampsia.

## Acknowledgements

We acknowledge all the pregnant women included in the present PreCAS study; the clinical staff enrolling patients at the Departments of Gynecology and Obstetrics, University Hospital of Southern Denmark, Esbjerg and Odense, Denmark and Kathrine Overgaard, Anette Larsen, Gunhild Nielsen and Lars Christian Nielsen, Unit for Thrombosis Research, Department of Regional Health Research, University of Southern Denmark, Esbjerg, Denmark for managing blood samples and biochemical analysis.

Author Contributions: J.B.G., J.J.S., A.C.G., J.S.J., and Y.P. designed and directed the research. A.C.G. collected and managed data in collaboration with B.B.D. A.C.G. conducted the statistical analyses. A.C.G. wrote the initial draft. J.J.S., J.B.G., and Y.P. developed methods for the study. All authors contributed to the article and approved the submitted version.

Funding information: Lida and Oskar Nielsens Foundation, Esbjerg Foundation, Gangsted Foundation, The Research Foundation of Southern Denmark and ‘Et Sundere Syddanmark’.

### Conflicts of interest

There are no conflicts of interest.
